# From framework to frontline: Embedding enterprise risk management into emergency department fall prevention

**DOI:** 10.1002/jhrm.70012

**Published:** 2025-09-17

**Authors:** Yalcin Golcuk, Ömer Faruk Karakoyun

**Affiliations:** ^1^ Department of Emergency Medicine, Faculty of Medicine Muğla Sıtkı Koçman University Muğla Turkey; ^2^ Emergency Medicine Service, Muğla Training and Research Hospital Muğla Turkey

## Abstract

This letter to the editor responds to Bailey and Delchamps’ recent article on integrating enterprise risk management (ERM) in fall‐related injury prevention. We extend their framework to emergency departments (EDs), emphasizing the strategic advantage of initiating individualized fall risk assessment at the point of triage. Many high‐risk indicators—such as anticoagulant use and cognitive impairment—are already accessible in ED settings and can be embedded into machine learning–supported tools like the Rothman index or fall triage score. We also highlight the financial implications of fall‐related injuries originating in or near the ED, noting their potential to increase hospital length of stay and trigger non‐reimbursable costs. The authors’ inclusion of risk matrices and heat maps presents scalable opportunities for safety prioritization in dynamic ED environments. We conclude by recommending prospective validation of ERM‐based approaches within level 1 trauma centers and invite collaboration to test the framework's effectiveness in real‐world emergency settings.

*Dear Editor*,

The recent article by Bailey and Delchamps provides a well‐structured and forward‐thinking framework for addressing inpatient fall‐related injuries through Enterprise Risk Management (ERM).^1^ While their approach centers on the inpatient setting, we wish to highlight its strong applicability to the emergency department (ED), where high‐risk patients are often first encountered. The strategic value of initiating injury risk stratification at the ED level has not been sufficiently explored in current literature and deserves focused attention.

The shift in focus from fall prevention to injury mitigation—anchored in individualized risk profiles—is a particularly valuable contribution. Many of the authors’ identified risk predictors, including oral anticoagulant use, cognitive impairment, polypharmacy, and urologic comorbidities, are commonly identifiable at the point of ED presentation. These variables are increasingly integrated into early warning systems and electronic health record–based decision tools.^2 ^Recent developments, such as machine learning–enhanced triage systems and predictive indices like the Rothman Index and the Fall Triage Score, have demonstrated promising results in early risk flagging.^3,4 ^Embedding these risk indicators into the ED triage workflow—supported by clinical decision support platforms—could enable timely upstream intervention before the patient transitions to inpatient care.

The financial and operational implications of fall‐related injuries that originate in or shortly after ED care are also critical. As the authors note, injuries from inpatient falls result in a >6‐day increase in length of stay and significantly higher hospitalization costs. For patients requiring surgical intervention, this impact is magnified.^5 ^These injuries are often classified as hospital‐acquired conditions, rendering them non‐reimbursable and exposing institutions to regulatory penalties and legal liability. Studies from the past five years have further reinforced the preventability of many such injuries through systems‐based approaches and targeted interventions.^6 ^ERM‐based tactics align with Step 4: strategic risk response, including early harm reporting, billing interventions (e.g., account suspension), and pharmacist‐led fall risk–increasing drug reviews. These measures may substantially mitigate institutional risk exposure in emergency settings.

Moreover, the authors’ inclusion of risk scoring matrices and heat map visualization presents an opportunity for adaptation in ED contexts. High‐frequency ED domains (e.g., ‘Staff Shortages,’ Risk Score 25 in Fig. 4) could be prioritized via heat map thresholds, guiding the allocation of limited safety resources toward the most impactful vulnerabilities. The framework's emphasis on evaluating mitigation efficacy through a Control Effectiveness Rating further enhances its relevance for dynamic, high‐acuity environments such as the ED.

We propose that future research validate the ERM model in high‐volume emergency settings, including Level 1 trauma centers. Prospective studies that measure fall‐related injury incidence, cost avoidance, and staff response time before and after ERM‐based implementation would be highly valuable in informing widespread adoption. We invite the authors to collaborate on validating their ERM framework in multicenter ED settings.

In conclusion, Bailey and Delchamps’ work adds conceptual clarity and strategic depth to fall‐related harm prevention. We believe that extending this model into emergency departments represents a logical and impactful next step—one that could enhance patient safety, reduce avoidable harm, and strengthen institutional resilience at the frontline of acute care.

## AUTHOR CONTRIBUTIONS

**Yalcin Golcuk**: Conceptualization; Writing—original draft; Writing—review and editing, **Ömer Faruk Karakoyun**: Conceptualization; Writing—original draft; Writing—review and editing.

## CONFLICT OF INTEREST STATEMENT

The authors declare that the article content was composed in the absence of any commercial or financial relationships that could be construed as a potential conflict of interest.

## References

[1] Bailey RO , Delchamps SL . Reducing the cost of inpatient falls: An ERM perspective. J Healthc Risk Manag . 2025. 10.1002/jhrm.70003 40405571

[2] Liu Q , Zhang Y , Sun J , et al. Early identification of high‐risk patients admitted to emergency departments using vital signs and machine learning. World J Emerg Med. 2025;16(2):113‐120. 10.5847/wjem.j.1920-8642.2025.031 40135217 PMC11930554

[3] Wengerter BC , Pei KY , Asuzu D , Davis KA . Rothman Index variability predicts clinical deterioration and rapid response activation. Am J Surg. 2018;215(1):37‐41. 10.1016/j.amjsurg.2017.07.031 28818297

[4] Mao A , Su J , Ren M , Chen S , Zhang H . Risk prediction models for falls in hospitalized older patients: a systematic review and meta‐analysis. BMC Geriatr. 2025;25(1):29. Published 2025 Jan 14. 10.1186/s12877-025-05688-0 39810076 PMC11730783

[5] Aryee E , James SL , Hunt GM , Ryder HF . Identifying protective and risk factors for injurious falls in patients hospitalized for acute care: a retrospective case‐control study. BMC Geriatr. 2017;17(1):260. Published 2017 Nov 7. 10.1186/s12877-017-0627-9 29115921 PMC5678557

[6] Morris ME , Webster K , Jones C , et al. Interventions to reduce falls in hospitals: a systematic review and meta‐analysis. Age Ageing. 2022;51(5):afac077. 10.1093/ageing/afac077 35524748 PMC9078046

